# Tracheobronchial Amyloidosis: A Rare Airway Disorder With Diagnostic and Therapeutic Challenges—A Case Report and Literature Review

**DOI:** 10.1002/rcr2.70171

**Published:** 2025-04-13

**Authors:** Filip Shterev, Vladimir Aleksiev, Veselin Chonov, Boyko Yavorov, Stanislav Kartev, Dimcho Argirov

**Affiliations:** ^1^ I‐st Department of Internal Diseases, Section of Pneumology and Phthysiatrics Medical University of Plovdiv Plovdiv Bulgaria; ^2^ Clinic of Thoracic Surgery Plovdiv University Multiprofile Hospital for Active Treatment Kaspela Plovdiv Bulgaria; ^3^ Department of Cardiovascular Surgery Medical University of Plovdiv Plovdiv Bulgaria; ^4^ Department of Clinical Pathology Plovdiv University Multiprofile Hospital for Active Treatment Kaspela Plovdiv Bulgaria; ^5^ Department of General and Clinical Pathology Medical University of Plovdiv Plovdiv Bulgaria; ^6^ Department of Special Surgery Medical University of Plovdiv Plovdiv Bulgaria

**Keywords:** airway obstruction, bronchoscopy, protein deposition, therapeutic management, tracheobronchial amyloidosis

## Abstract

Tracheobronchial amyloidosis (TBA) is a rare, localised form of amyloidosis characterised by the extracellular deposition of abnormal proteins within the tracheal and bronchial tissues. This condition, although uncommon, can significantly impact airway function, leading to symptoms such as persistent cough, dyspnea and airway obstruction. This report highlights the clinical presentation, diagnostic approaches and therapeutic options for TBA, emphasising the need for individualised management strategies and comprehensive patient care. This case describes a 62‐year‐old male with a history of smoking debuting with progressive hoarseness, dyspnea and dysphagia, who was initially diagnosed with tracheobronchial amyloidosis following biopsy of a subglottic mass. After surgical excision and a 3‐year disease‐free interval, he experienced multiple recurrences requiring further interventions, including bronchoscopic evaluation. Histopathological confirmation of recurrent amyloidosis led to a decision for strict follow‐up, as symptoms remained mild post‐biopsy.

## Graphical abstract

This report highlights the clinical presentation, diagnostic approaches and therapeutic options for tracheobronchial amyloidosis (TBA), emphasising the need for individualised management strategies and comprehensive patient care.

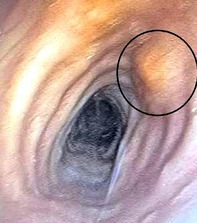

## 1 Introduction

Tracheobronchial amyloidosis (TBA) is a rare idiopathic group of disorders characterised by an abnormal extracellular deposition of autologous proteins within the bronchial tree. This ultimately leads to impaired organ function because of tracheobronchial stenosis and occlusion [1]. Isolated cases of amyloidosis, limited specifically to tracheal and bronchial tissue, are a notably rare occurrence. Diseases caused by abnormal amyloid deposits are generally classified as primary and secondary, depending on whether the condition is associated with any coexisting disorders such as chronic inflammation. Further subtypization recognises local and systemic amyloidosis, based on its distribution [2].

Recent studies have further highlighted the different histological subtypes of amyloidosis and their implications in airway involvement. AL amyloidosis, associated with immunoglobulin light chains, is the most common subtype affecting the tracheobronchial tree, whilst AA amyloidosis, secondary to chronic inflammation, rarely involves the airways [2]. Emerging data suggest a potential but under‐reported role of ATTR amyloidosis (transthyretin‐related deposits) in tracheobronchial manifestations, warranting further investigation [3].

Pulmonary involvement has an estimated incidence between 5 and 10 cases per million persons/year [3]. Three different forms of pulmonary amyloidosis are recognised, ranging from nodular pulmonary amyloidosis and tracheobronchial amyloidosis to diffuse alveolar‐septal amyloidosis. Tracheobronchial involvement accounts for 25%–50% of localised pulmonary amyloidosis [4]. Nodular pulmonary amyloidosis presents as localised nodular amyloid deposits and is considered harmless, whilst diffuse parenchymal amyloidosis is associated with systemic disease [5]. Tracheobronchial involvement occurs in approximately 25%–50% of localised pulmonary amyloidosis cases, predominantly of the AL (light‐chain) subtype [6].

Many hypotheses try to explain amyloidogenesis. Some studies emphasise the role of mutations in thermodynamic and kinetic pathways [6], whilst other researchers theorise that non‐physiological proteolysis and deficient physiological proteolysis have a part to play in the systemic precipitation and formation of these aberrant proteins [7]. The cascade, which leads to amyloid accumulation, requires the involvement of precursor proteins, which are then misfolded and undergo a process of nucleation, polymerisation, fibre elongation and tissue deposition [8]. Unlike systemic amyloidosis, localised TBA lacks plasma cell dyscrasias, further distinguishing it from AL amyloidosis and underscoring its unique pathophysiology.

The clinical manifestation of tracheobronchial amyloidosis is highly variable depending on the severity of the disease. Patients typically present with persistent cough, dyspnea and, in some cases, hemoptysis or stridor [9]. Typically, the disease remains asymptomatic, or the existing symptoms are non‐specific. They may also include hoarseness, dyspnea and dysphagia [10].

## 2 Case Report

**FIGURE 1:**
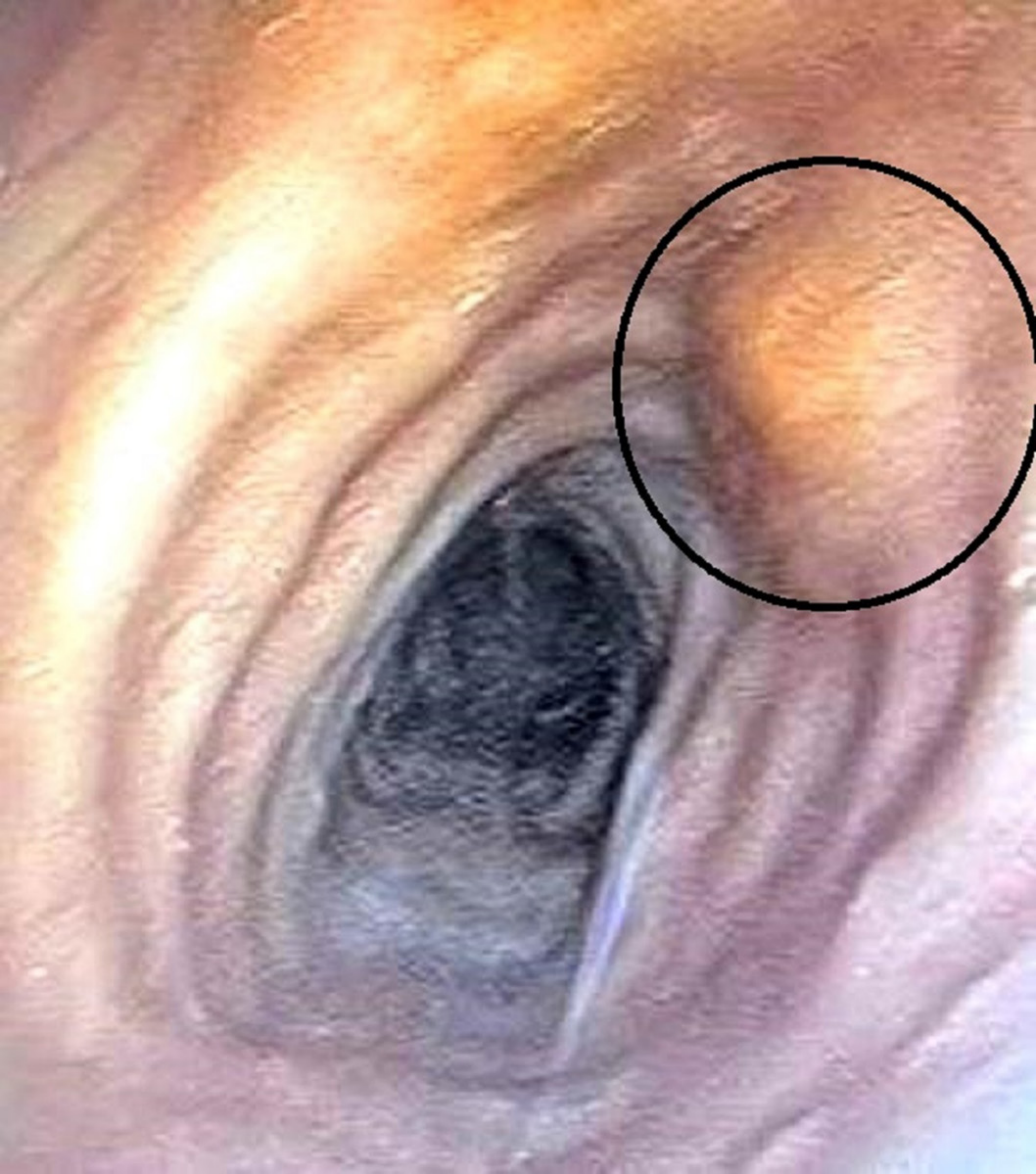
This image shows the upper third of the trachea. The highlighted abnormality consists of a raised bump, localised within the tracheal mucosa, which is consistent with amyloid deposits.

**FIGURE 2:**
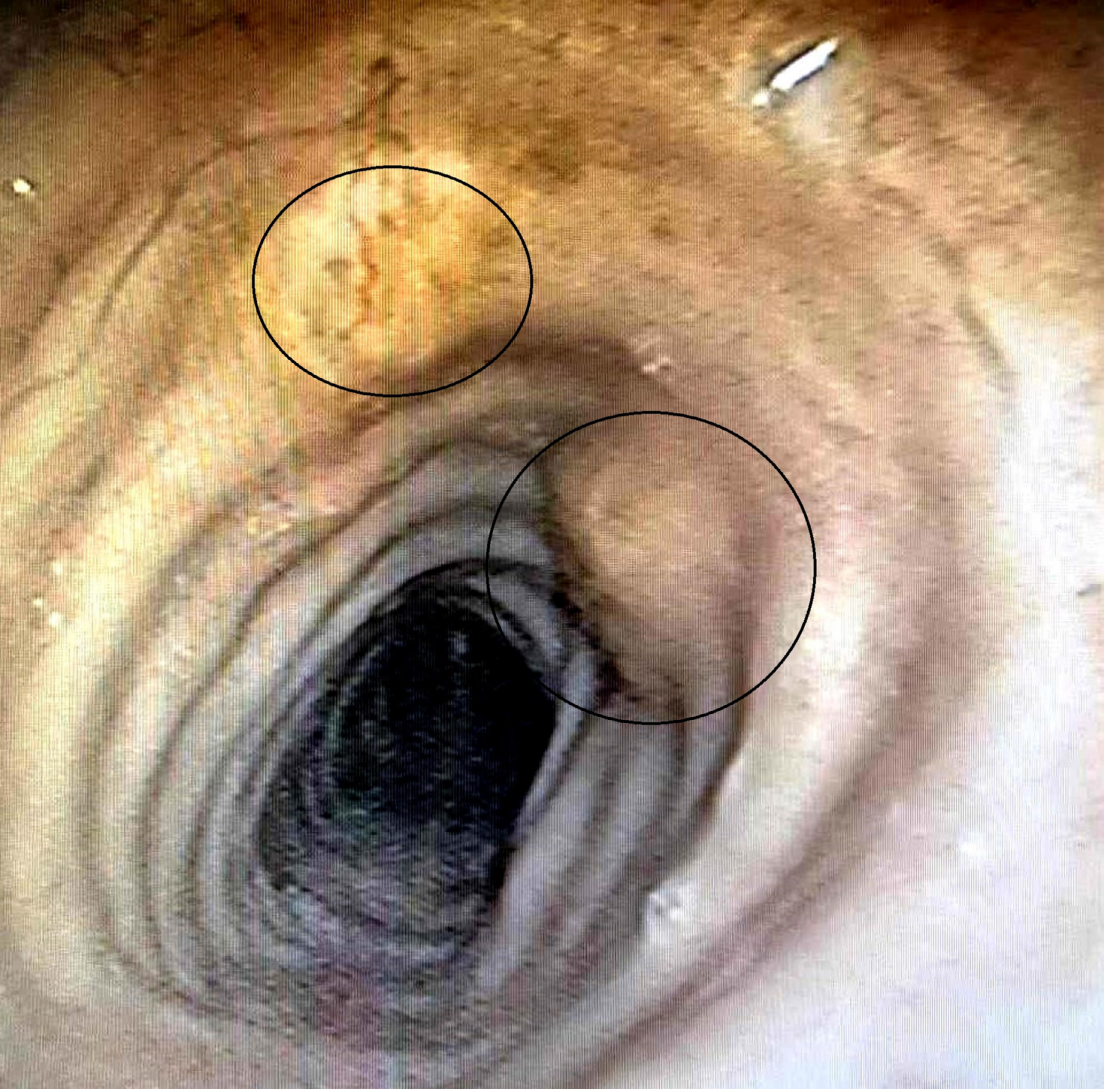
This image shows the middle tracheal segment with amyloid deposits manifesting as irregular protrusions within the airway lumen.

**FIGURE 3:**
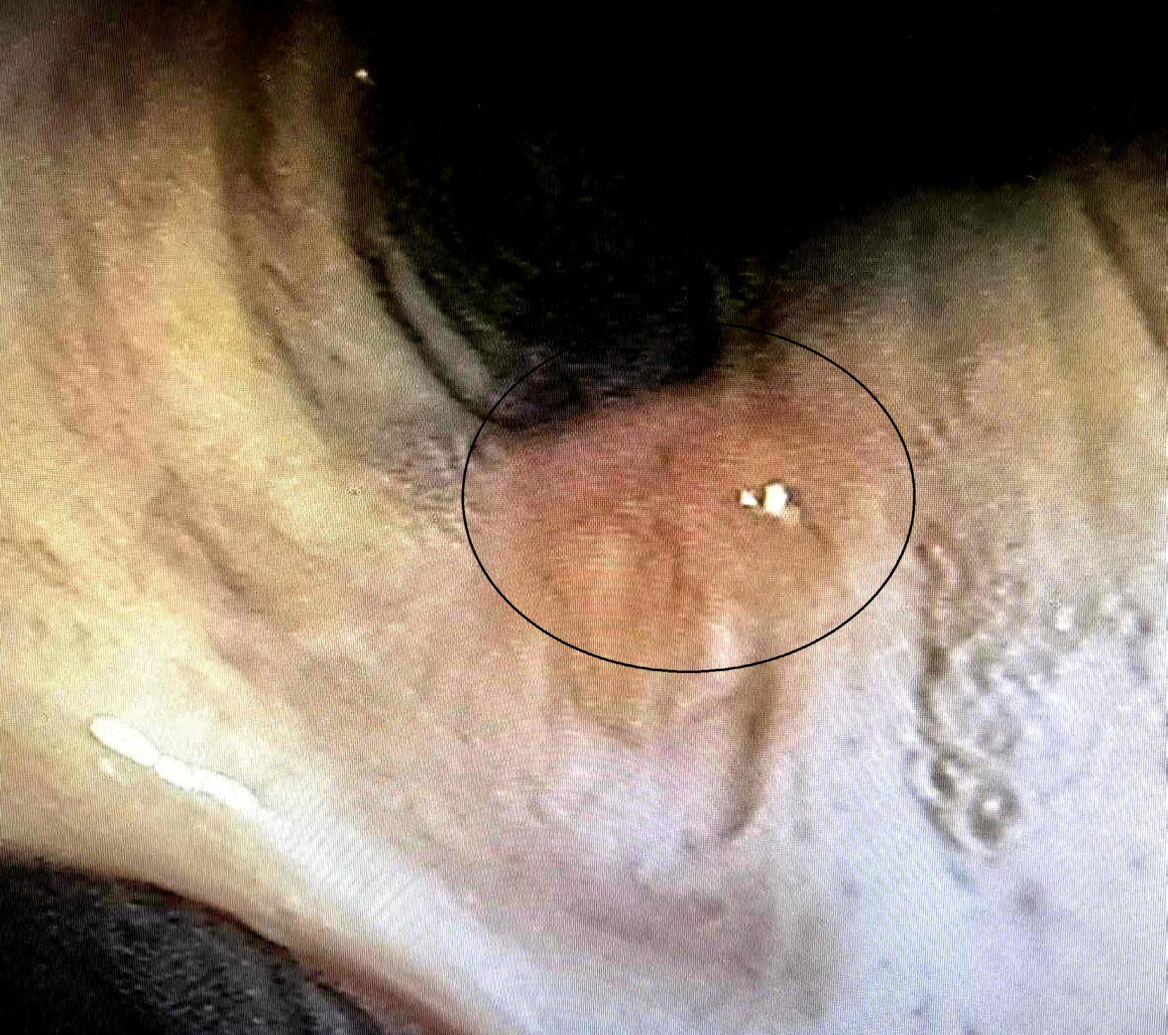
This image captures the tracheal bifurcation (carina). The raised lesion, characteristic of an amyloid deposit, is prominent on the mucosal surface near the bifurcation.

A 62‐year‐old male patient first presented to the Clinic of Otorhinolaryngology with a 6‐month history of gradually worsening hoarseness and dyspnea upon exertion. He had also reported considerable difficulty swallowing solid foods as well as a perceived foreign body sensation in the pharynx. He was a past smoker with a combined total of 40 pack‐years. No past medical conditions or comorbidities were noted. Flexible laryngoscopy revealed a submucosal multifocal subglottic mass. A decision was made for the patient to undergo micro‐direct laryngoscopy. Intraoperatively, after visualising the diffuse subglottic mass, the glottis was observed as significantly narrowed. Several biopsies were taken for frozen section. Initial frozen section pathology was consistent with tracheobronchial amyloidosis. After diagnosis, a full excision with subsequently confirmed clear margins was carried out. The patient experienced a 3‐year disease‐free period, after which he was admitted with identical complaints and was diagnosed with recurrent amyloidosis of the glottis, necessitating a second excisional biopsy to be carried out. A third less severe relapse occurred 2 years after the second surgical intervention, raising suspicion of an underlying condition affecting the trachea or distal airway. The patient was then referred to the clinic of thoracic surgery in order to undergo a complete evaluation of the tracheobronchial tree. Firstly, computed tomography was done which revealed preserved parenchymal transparency with solitary areas of pleural thickening, but no infiltrative or nodular lesions. The tracheal and bronchial lumen seemed unobstructed. His bloodwork and pulmonary function tests were unremarkable as well. A videobronchoscopy revealed two yellowish polypoid formations which are displayed on Figures 1 and 2 in the upper third of the membranous portion of the trachea. Samples were taken for histology. An area resembling a granular formation was found and biopsied on the right wall of the trachea just above the carina as seen on Figure 3. The left and right bronchi were examined to the subsegmental level with normal mucosa and moderate amounts of serous secretions. The patient underwent an uneventful periprocedural period and was discharged on the next day after the intervention.

**FIGURE 4:**
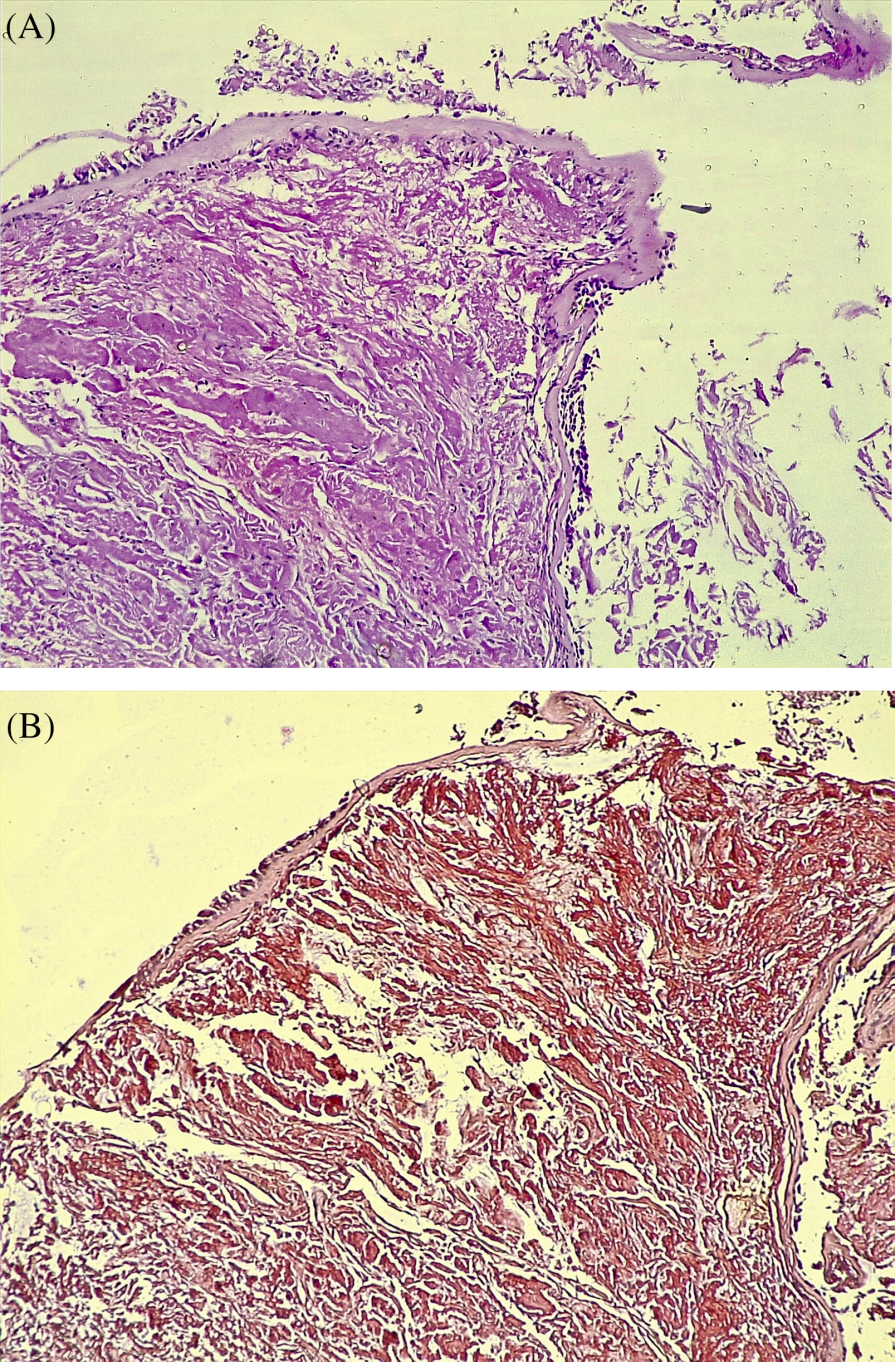
(A) A pinch biopsy from the wall of the bronchus with preserved covering cylindrical epithelium. Subepithelial abnormal deposition of homogeneous pink matter, at first as pinkish red streaks between normal structures. Later, atrophy occurs, and they are completely displaced by the deposition of amyloid. It is proven by staining with Congo Rot, which stains amyloid deposits in pink or red under a conventional light microscope. (B) Histological section stained with Congo red under light microscopy displaying amyloid deposits (pink‐red areas).

Histopathological analysis of the biopsies taken confirmed the diagnosis of tracheobronchial amyloidosis in all three biopsied specimens (Figure 4).

To exclude generalised involvement of amyloidosis, including the heart and kidneys, we conducted a thorough diagnostic workup. For cardiac assessment, we performed electrocardiography (ECG), which showed no significant low‐voltage QRS complexes or pseudo‐infarction patterns. An echocardiogram was performed, revealing no evidence of left ventricular hypertrophy, diastolic dysfunction, or granular sparkling suggestive of amyloid infiltration. We measured serum cardiac biomarkers, including troponins and natriuretic peptides, which were within normal limits, excluding subclinical cardiac involvement.

For renal involvement, kidney function tests, including serum creatinine, estimated glomerular filtration rate (eGFR) and urinalysis for proteinuria, were within normal ranges. Immunofixation of serum and urine proteins showed no monoclonal gammopathy and serum kappa/lambda free light chain ratio was normal, ruling out AL amyloidosis. These comprehensive evaluations confirmed that amyloid involvement was localised to the trachea without generalised or systemic organ involvement.

To exclude systemic autoimmune vasculitis as a potential cause, we tested for anti‐neutrophil cytoplasmic antibodies (ANCA), including perinuclear ANCA (pANCA) and cytoplasmic ANCA (cANCA). Both tests returned negative, ruling out associated conditions such as granulomatosis with polyangiitis (cANCA) or microscopic polyangiitis (pANCA).

Due to the mild severity of symptoms and lack of generalised involvement, it was decided for the patient to maintain a strict follow‐up. He remained asymptomatic a full month after the biopsy and was planned for a second bronchoscopy and CT evaluation at the third month of diagnosis.

## 3 Discussion

Tracheobronchial amyloidosis, occurring exclusively in the tracheal and bronchial tissue, is an unusual finding, with only a few hundred cases being described in the available literature [11, 12, 13, 14, 15, 16, 17, 18, 19]. Computed tomography is an integral part of the diagnostic work‐up of this disease. There are a few distinctive findings, which are often characteristic. Mucosal abnormalities have been described, ranging from mucosal thickening, with or without calcification [20] to solitary lesions, mimicking malignancy [21]. Most commonly, tracheobronchial amyloidosis remains localised with no systemic or parenchymal involvement [22].

Videobronchoscopy is the gold standard for the diagnosis, treatment and follow‐up of tracheobronchial amyloidosis. Upon initial inspection, amyloid deposits appear as brittle, pale and oedematous areas of bronchial mucosa [23]. Histopathological examination after Congo red staining reveals a squamous epithelium with subepithelial deposits of pink hyaline extracellular material, which is pathognomonic [24]. In recent years, endobronchial ultrasound (EBUS) has emerged as a valuable tool for deeper tissue characterisation, aiding in both diagnosis and biopsy guidance.

A comprehensive study, conducted by Smesseim [25] showcases several treatment modalities and highlights the most prevalent treatment options. The most common approach included therapeutic bronchoscopy (30.3%), which involved Nd: YAG laser irradiation, mechanical debulking, argon plasma therapy, stent placement, balloon dilatation, cryotherapy and intermittent microwave ablation. External beam radiotherapy was chosen in 22% of the cases, and a significant number of patients were managed with the wait‐and‐see approach (21.7%). A small number of patients received corticosteroids, colchicine, melphalan and rituximab. Just 4% of patients required surgery after experiencing severe respiratory distress. Therapeutic interventions remain highly individualised, with recent reviews reporting that 30.3% of patients undergo therapeutic bronchoscopy, including Nd:YAG laser, cryotherapy and mechanical debulking. Radiotherapy is employed in 22% of cases, whilst 21.7% of patients are managed conservatively with close follow‐up [26]. Immunomodulatory therapies such as colchicine, corticosteroids and chemotherapy (melphalan, rituximab) have been explored, though their efficacy remains inconsistent. Long‐term prognosis varies, with a 5‐year mortality rate ranging from 30% to 50%, predominantly due to airway obstruction.

The long‐term prognosis of tracheobronchial amyloidosis largely depends on the spread of the disease and accompanying conditions. It is theorised in numerous reports that this pathology is associated with Sjogren's Syndrome, which also makes management even more difficult [26]. Furthermore, comorbidities, such as bronchial asthma and COPD, additionally aggravate symptoms [27]. A study carried out by states that the mortality rate for tracheobronchial amyloidosis falls around 30% after 7–12 years after diagnosis [28]. Major airway obstruction seems to be the leading cause of death, with another study describing a 5‐year mortality rate of 30%–50%, especially in patients with diffuse tracheobronchial amyloidosis [29].

In conclusion, the presented case is notable for its recurrent nature over a prolonged period (three recurrences over 5 years), requiring diverse therapeutic interventions including multiple bronchoscopic evaluations and surgical excisions. Such recurrent presentations with stable and mild symptoms post‐intervention are rare in the literature.

Tracheobronchial amyloidosis is an unusual form of localised amyloidosis, affecting the trachea and distal bronchi. It exclusively affects the tracheobronchial tissue and rarely manifests as a systemic disease. Clinical suspicion should be raised in patients with symptoms of airway obstruction and distinct findings upon CT evaluation. Tissue biopsy via bronchoscopic examination, coupled with a thorough histopathological examination, utilising the Congo red stain, is the only way to achieve a definitive diagnosis. The treatment in tracheobronchial amyloidosis is varied and targets current symptoms. A thorough follow‐up is mandatory with patients undergoing frequent scans and re‐evaluation bronchoscopies in order to prevent complications and critical airway stenosis.

## Author Contributions

**Filip Shterev:** conceptualisation, methodology, data analysis, and manuscript writing. Responsible for the overall study design and ensuring the accuracy of the data presented. **Vladimir Aleksiev:** literature review, data collection, and interpretation of results. Contributed to manuscript revisions and provided critical feedback. **Veselin Chonov:** data collection, drafting sections of the manuscript, and reviewing the final manuscript for intellectual content. **Boyko Yavorov:** supervision of research and manuscript revision. Ensured that all ethical guidelines were followed throughout the study. **Stanislav Kartev:** data analysis and manuscript writing. Assisted in study design and interpretation of the findings. **Dimcho Argirov:** literature review, data collection, and contribution to manuscript revisions.

## Ethics Statement

Specific written informed consent for the publication of this manuscript has been obtained from the patient involved in this study.

## Conflicts of Interest

The authors declare no conflicts of interest.

## Data Availability

The data that support the findings of this study are available on request from the corresponding author. The data are not publicly available due to privacy or ethical restrictions.
